# Supplementary material for: Lactobacillus helveticus HY7804 Modulates the Gut–Liver Axis to Improve Metabolic Dysfunction-Associated Steatotic Liver Disease in a Mouse Model
Source: Int J Mol Sci. 2025 Apr 10;26(8):3557. doi: 10.3390/ijms26083557 (PMC12027198; doi:10.3390/ijms26083557)
Supplement: Supplementary file 1 [file ijms-26-03557-s001.zip › ijms-3543667-supplementary.pdf]

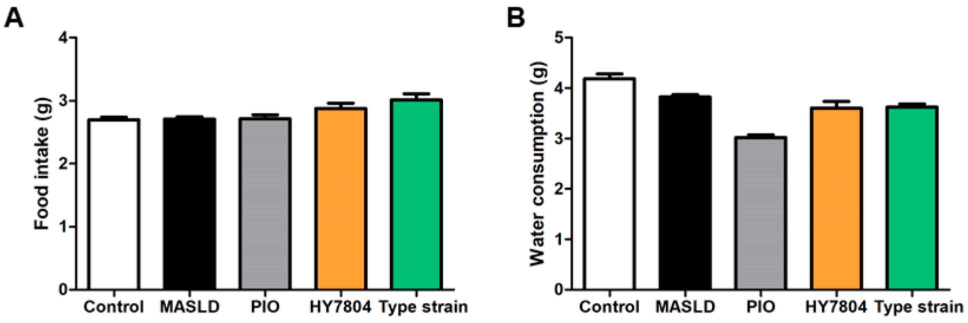

Figure S1. Dietary intake and water drinking (*n*=7 mice per group). (A) Amounts of dietary intake (g), (B) water consumption (g) during animal experiments. Results are presented as the mean ± SD.

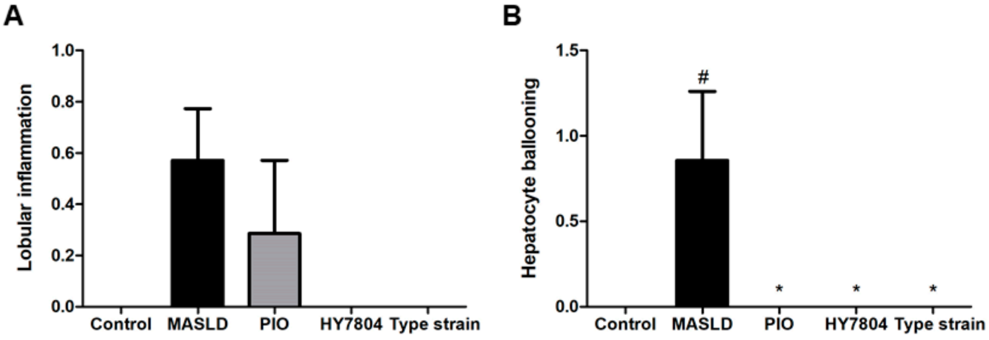

Figure S2. Hepatic histological analysis (*n*=7 mice per group). (A) Score of lobular inflammation (g), (B) Score of hepatocyte ballooning. Results are presented as the mean ± SD. #p<0.05 vs. Control group, * p < 0.05 vs. MASLD group (one-way ANOVA with post-hoc analysis). MASLD, mice fed an MASLD-inducing diet; PIO, pioglitazone + MASLD; HY7804, *Lactobacillus helveticus* HY7804 + MASLD; Type strain, *Lactobacillus helveticus* type strain KCTC 3545 + MASLD.

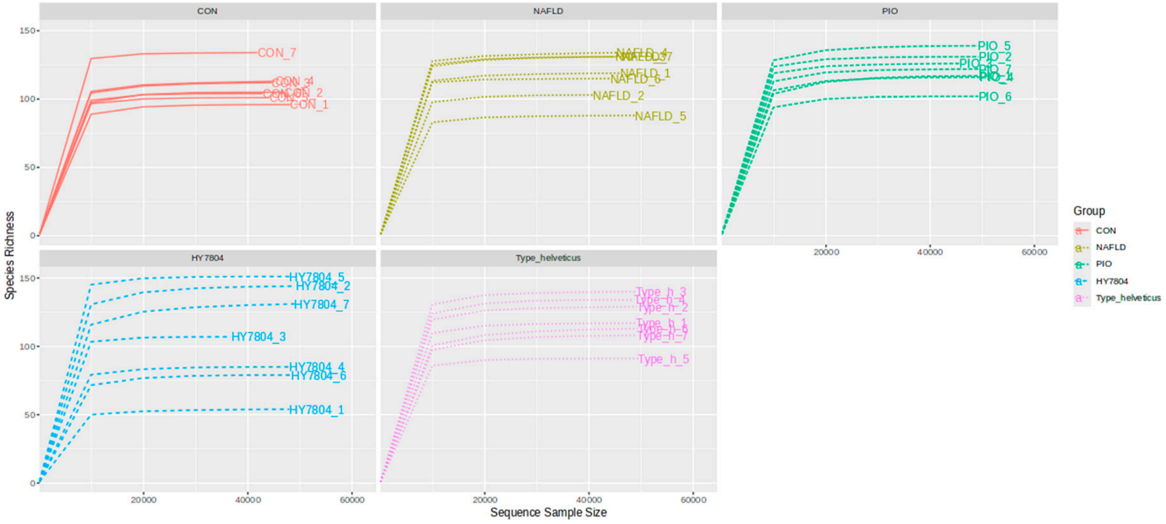

Figure S3. Rarefaction curve (*n*=7 mice per group).
